# Effects of probiotics on the lipid profile: systematic review

**DOI:** 10.1590/1677-5449.180124

**Published:** 2019-08-09

**Authors:** Carlos Jorge Maciel Uchoa Gadelha, Alane Nogueira Bezerra

**Affiliations:** 1 Faculdade Metropolitana da Grande Fortaleza – FAMETRO, Fortaleza, CE, Brasil.; 2 Universidade Federal do Ceará – UF, Programa de Pós-graduação em Ciências Médicas, Fortaleza, CE, Brasil.

**Keywords:** dyslipidemias, hypercholesterolemia, probiotics

## Abstract

Alterations in the intestinal microbiota can modulate mechanisms involving risk factors for cardiovascular diseases, including dyslipidemias The objective was to review the effects of probiotic supplementation on the prevention and treatment of changes to the lipid profile. The searches were run on the PubMed database, using the descriptors “probiotics and lipid profile” and “probiotics and dyslipidemia,” with publication dates restricted to 2013 to 2018. Supplementation with probiotics significantly reduced total cholesterol, LDL-c, and triglycerides and increased HDL-c. Some benefits were observed on anthropometric variables, glycemic control, oxidative stress, inflammation, and immune system. The present study suggests that probiotic supplementation should be indicated as adjunctive treatment for dyslipidemias. Further studies should be developed to clarify long-term effects, as well as the influence of probiotics in combination with drug therapy.

## Introduction

The process of urbanization and the lifestyle changes that result have contributed to a substantial increase in the rates of chronic diseases, in particular of cardiovascular diseases (CVDs). These are the number one cause of death worldwide and are responsible for considerable economic losses and significant healthcare expenditure.^1^ Build-up of fat, primarily visceral fat, occurs through complex interactions between genetics and environmental factors and is associated with subclinical systemic inflammation and with many different risk factors for development of CVDs.^2^ Among these risk factors, hypercholesterolemia, hypertriglyceridemia, increases in the concentrations of low density lipoproteins (LDL), and reductions in the levels of high density lipoproteins (HDL) are all important targets for attempts to prevent CVDs.^3^^,^ 4

The intestinal microbiota (IM) is responsible for many different biochemical reactions and is considered an important regulator of metabolic status.^5^ According to Huttenhower et al.,^6^ the IM is directly linked with maintenance of good health, whether intestinal or systemic. Microbiota instability, known as dysbiosis, can directly affect the emergence and resulting complications of many different diseases, especially non-transmissible chronic diseases. Changes in the composition and function of IM occur in metabolic syndrome and in CVDs, both of which are conditions in which dyslipidemia may be present.

People with hypercholesterolemia have lower bacterial diversity in their IM when compared with controls. Additionally, the profile of the microorganisms that are present is different, which suggests that IM possibly play a role in development of hypercholesterolemia.^7^ Manipulation of the IM with probiotics results in several benefits for the host.^8^^,^ 9 Probiotics are already in use in human medicine, both for disease prevention and for treatment, by controlling the IM.^10^

The hypocholesterolemic effects of probiotics have been investigated both in vitro and in vivo, although conflicting results have been observed.^11^ Therefore, in view of the need for, and importance of, new therapeutic methods to control and improve patients’ lipid profiles to help with treatment of dyslipidemia and other non-transmissible chronic diseases, the objective of this study was to review the effects of supplementation with probiotics for prevention and treatment of lipid profile abnormalities.

## Methods

This is a systematic review based on searches of the PubMed databases using a combination of the descriptors “probiotics and lipid profile” and “probiotics and dyslipidemia,” with publication dates restricted to January 2013 to March 2018. Clinical trials involving subjects over the age of 18 and published in English were included in the review. The exclusion criteria were studies involving pregnant women and breastfeeding mothers.

## Results

**Figure 1:**
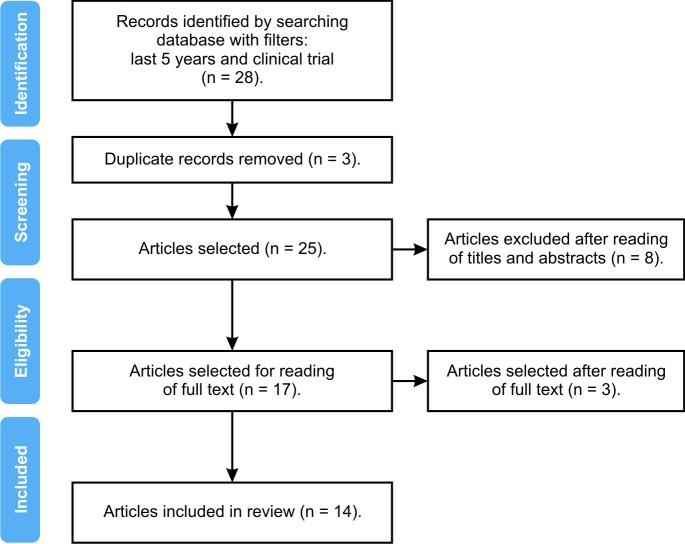
Flow diagram illustrating selection of articles for the review.

A total of 28 full text articles describing clinical trials were identified. After applying the exclusion criteria and eliminating studies undertaken with pregnant women and breastfeeding mothers, removing duplicates, and reading titles, abstracts, and full texts, 14 clinical trials were selected for inclusion. The Preferred Reporting Items for Systematic Reviews and Meta-Analyses (PRISMA) method flow diagram was used to document the details of the article selection process (Figure 1).

The articles selected analyzed the influence of probiotics on the lipid profile, as summarized in Table 1.^12^^-^ 25 However, not all of the studies evaluated these markers using primary methods.^15^^,^ 16 ^,^ 17 ^,^ 22

**Table 1:** Summary of the articles reviewed.

**Authors/Type of study**	**Description of samples**	**Objectives**	**Results**	**Limitations**	**Conclusions**
Ahn et al. (2015)^12^/Clinical trial.	121 non-diabetic people with hypertriglyceridemia.	To investigate the effects of supplementation with two strains, *Lactobacillus curvatus HY7601* and *L. plantarum KY1052*, on capacity for reduction of TG in non-diabetics with borderline and moderate hypertriglyceridemia.	Between the groups, there was a 18.3% reduction in TG and a 15.6% reduction in LDL particle size and a 21.1% increase in apo A-V (p*<*0.05*).* TG and apo A-V values were inversely correlated.	Short duration, small sample, no control of diet, and no analysis of the IM.	The authors concluded that supplementation with these strains reduced serum TG levels, and the greatest effect was observed in subjects with higher levels.
	Intervention period: 12 weeks.				
	IG: 2 grams of powder containing *Lactobacillus curvatus HY7601* and *L. plantarum KY1052*, 0.5 x 10^10^ CFU of each.				
	PG: 2 grams of powder, with no microorganisms				
Bernini et al. (2016)^13^/Clinical trial.	51 people with metabolic syndrome aged from 18 to 60 years.	To assess the influence of milk fermented with *Bifidobacterium lactis* HN019 on the lipid profile, glycemic control, and inflammatory profile of patients with metabolic syndrome.	Significant reductions were observed in TC (p=0.009) and LDL-c (p=0.008).	Short duration, small sample, and no analysis of the IM.	The data showed potential effects of *Bifidobacterium lactis* HN019 on reduction of lipids in the blood.
	Intervention period: 45 days.				
	IG: 26 subjects, consumed milk fermented with 2.72 x 10**^10^** CFU of *Bifidobacterium lactis HN019*.				
	PG: no intervention.				
Cavallini et al. (2016)^14^/Clinical trial.	49 hypercholesterolemic males aged 45 to 48 years.	To investigate the influence of a soy product fermented with *Enterococcus faecium* CRL 183 and *Lactobacillus helveticus* 416 plus isoflavones.	Only subjects in G1 exhibited a significant reduction in TC. When compared with G2, the result at the end of the study was reduction in LDL-c (p<0.05) in both treatment groups, from days 30 to 42, and also in the TC/HDL ratio.	Short duration, small sample, and no analysis of the IM.	Regular consumption of the probiotics *Enterococcus faecium* CRL 183 and *Lactobacillus helveticus* 416 contributed to a reduction in markers of cardiovascular risk in hypercholesterolemic men, with improved lipid profile and reduced oxidation of LDL particles.
	Intervention period: 42 days.				
	G1: 200 mL of soy product fermented with the probiotic *Enterococcus faecium* CRL 183 and *Lactobacillus helveticus* 416, supplemented with isoflavone.				
	G2: 200 mL unfermented soy product.				
Childs et al. (2014)^15^/Clinical trial.	43 healthy subjects, aged from 25 to 65 years.	To investigate the effects of xylo-oligosaccharide and *Bifidobacterium animalis* BI–07 on the IM and on immune function.	Higher HDL levels were observed in subjects given xylo-oligosaccharide, with no difference in the other plasma lipid levels tested.	Short duration and small sample.	The data indicate potential benefits of the xylo-oligosaccharide and *Bifidobacterium animalis* BI–07 on the capacity to increase HDL.
	Four different treatments were tested (G1: maltodextrin; G2*: Bifidobacterium animalis* BI–07, 10^9^ CFU; G3: xylo-oligosaccharide, 8 g/d; G4: *Bifidobacterium animalis* BI–07, 10^9^ CFU + xylo-oligosaccharide, 8 g/d).				
	Intervention period: 21 days, followed by 28 days’ washout.				
Dong et al. (2013)^16^/Clinical trial.	30 healthy volunteers aged 55 to 74 years.	To test probiotics on the immune systems of people with immunosenescence.	The data indicated that there was no significant reduction in TC or TG..	Short duration, small sample, lack of data on the lipid profile, and no analysis of the IM.	Consumption of the probiotic *Lactobacillus casei Shirota* did not have a significant effect on the lipid profile.
	Intervention period: 4 weeks.				
	IG: *Lactobacillus casei shirota* with 1.3 x 10^10^ CFU/day.				
	PG: skimmed milk without the probiotic.				
Dönmez et al. (2014)^17^/Clinical trial.	18 sedentary males with a mean age of 33.66 years.	To investigate the effects of koumiss as a probiotic, in combination with physical exercise, on hematological and biochemical variables in sedentary people	TC and TG tended to reduce in all groups, but only in G2 at day 15. HDL increased in all groups, and the greatest effect was observed in G2	Short duration, small sample, and no analysis of the IM.	The findings suggest that koumiss plus physical exercise provoked an increase in HDL.
	Intervention period: 15 days.				
	G1: koumiss;				
	G2: koumiss plus physical exercise.				
	G3:physical exercise.				
	Main probiotics used to ferment koumiss: *L. delbrueckii* subsp. *Bulgaricus, L. salivarus, L. buchneri, L. plantarum, L. casei, L. helveticus,* and *L. fermentum.*				
Fuentes et al. (2013)^18^/Clinical trial.	60 hypercholesterolemic subjects, aged 18 to 65 years.	To evaluate the effects of the AB-LIFE® formula on concentrations of lipids and on other parameters related to cardiovascular risk in hypercholesterolemic subjects.	The intervention significantly reduced TC and caused a trend to reduction of LDL-c and OX-LDL, compared with the PG. In the IG, there were significant (p<0.05) reductions in TC, LDL-C, LDL-C:HDL-C ratio, and OX-LDL (13.6%, 14.7%, 19.7%, and 13.6%, respectively), in relation to baseline. The increase in HDL-C (p<0.05) was only observed in the IG.	Short duration, small sample, and no analysis of the IM.	Supplementation with *Lactobacillus plantarum* CECT (7527, 7528, and 7529) made a significant contribution to reducing serum cholesterol in hypercholesterolemic patients, exhibiting better effects in those with higher cholesterol levels.
	BMI: 19-30 kg/m^2^.				
	Intervention period: 12 weeks.				
	IG: 1 capsule of *Lactobacillus plantarum* CECT (7527, 7528 and 7529) containing a 1.2 x 10^9^ CFU dose.				
	PG: 1 capsule containing no bacteria.				
Gomes et al. (2017)^19^/Clinical trial.	43 women, aged 20 to 59 years, with BMI in the range 24-40 kg/m^2^.	To investigate whether a mix of probiotics has additional effects, when compared with a dietary intervention alone, on body composition, lipid profile, endotoxemia, inflammation, and antioxidant and anti-inflammatory profiles.	There was no difference between groups in LDL-c, just a reduction in results for polyunsaturated fatty acids (PG= -5.65% vs. IG = - 18.63%, at p<0.04).	Short duration, small sample, and no analysis of the IM.	Supplementation with the mixture of probiotics had an additional effect when compared with the group with only a dietary intervention.
	Intervention period: 8 weeks.				
	IG: dietary intervention plus mixture of *Lactobacillus acidophilus*e, *L. casei*; *Lactococcus lactis*; *Bifidobacterium bifidum* and *lactis*, at a dosage of 2 × 10^10^.				
	PG: Dietary intervention only.				
Ivey et al. (2015)^20^/Clinical trial.	156 people with metabolic syndrome and mean age of 67 years.	To determine the effect of *Lactobacillus acidophilus La5* and *Bifidobacterium animalis*, subspecies *lactis Bb12*, in the form of yoghurt or capsules, on blood pressure and the lipid profile in men and women with metabolic syndrome.	There were no differences in lipid profile markers between groups (p<0.05).	Short duration, small sample, and no analysis of the IM.	The probiotic strains *Lactobacillus acidophilus La5* and *Bifidobacterium animalis*, subspecies *lactis Bb12* did not influence changes in lipid profile parameters, probably because baseline cholesterol values were relatively good.
	Intervention period: 6 weeks.				
	G1: yoghurt, plus placebo capsule; G2: probiotic capsule plus milk; G3 and G4: placebos.				
	Subjects who consumed the probiotic ingested at least 3 x 10^9^ CFU/day.				
	*Lactobacillus acidophilus La5* and *Bifidobacterium animalis*, subspecies *lactis Bb12* were used.				
Kullisaar et al. (2016)^21^/Clinical trial.	45 healthy volunteers aged 50 to 75 years, with BMI from 24-30 kg/m^2^ and borderline risk factors for cardiovascular disease.	To determine whether the special formulation *Reg’active Cholesterol*® has a positive effect on the cardiovascular system, lipid and inflammatory profiles, and glycated hemoglobin.	All participants exhibited significant reductions in LDL-c, TC, TG, and OX-LDL (p<0.05) and a tendency to improvements in HDL.	Short duration, no control group, small sample, no dietary control before or after the intervention, no analysis of the IM, and use of a compound formula containing several nutrients.	Consumption of the formula by people with borderline cardiovascular risk values over a 4-week period had a positive effect on reduction of LDL-c, TC, TG and OX-LDL.
	Intervention period: 4 weeks.				
	IG: 2 capsules per day containing the probiotic *L. fermentum* ME-3 (6 x 10^9^ CFU/day), plus other compounds.				
Ogawa et al. (2014)^22^/Clinical trial.	20 people with a mean age of 51.1 years and hypertriglyceridemia.	To examine the effects of the probiotic *Lactobacillus gasseri* SBT2055 (LG 2055) on postprandial response of blood lipids in Japanese subjects with hypertriglyceridemia.	With relation to fasting parameters, only non-esterified fatty acid levels exhibited a significant reduction (p<0.01). There was no significant difference in TG, TC, HDL, or LDL.	Short duration, small sample, and no analysis of the IM.	Consumption of the probiotic reduced non-esterified fatty acid levels after an oral fat overload and during the post-ingestion period of the probiotic, after 4 weeks consuming the microorganism.
	Intervention period: 4 weeks.				
	IG: 5 x 10^10^ CFU/day of *Lactobacillus gasseri* SBT2055 (LG 2055), with a frequency of 2 times a day.				
Rajkumar et al. (2014)^23^/Clinical trial.	60 overweight people aged 40 to 60 years.	To investigate whether probiotics, alone or in combination with omega 3, provoke any improvement in the lipid profile, insulin sensitivity, or inflammatory response in healthy overweight people.	G1: HDL increased by 18.5% (p<0.01), and LDL (p<0.05), TG, and VLDL (p<0.01) values reduced by 7.04%, 5.8%, and 12.98, respectively.	Short duration, small sample, difficult to identify the effect of any specific strain	An increase in HDL and reductions in TG, LDL, and VLDL were observed and the best results were observed in G3.
	Intervention period: 6 weeks.		G2: HDL increased by 23.2% and LDL reduced by 10.7%, TG reduced by 7.78%, and VLDL reduced by 7.78% (p<0.01).		
	G1: 112.5 x 10^9^ CFU with: *Bifidobacterium longum, Bifidobacterium infantis, Bifidobacterium breve, Lactobacillus acidophilus, Lactobacillus paracasei, Lactobacillus delbrueckii*, subspecies *bulgaricus*, *Lactobacillus plantarum,* and *Streptococcus salivarius subspecies thermophilus.*				
	G2: omega 3: 1 capsule with 180 mg of EPA and 120 mg of DHA.		G3: TC, TG, LDL, and VLDL reduced and HDL increased by 6.7% (p<0.01).		
	G3: probiotics and omega 3.				
Ryan et al. (2015)^24^/Clinical trial.	11 hypercholesterolemic men, aged 21 to 69 years.	To collect evidence on the effects of the probiotic *Saccharomyces boulardii* on the lipid profile and other markers in hypercholesterolemic adults.	Compared with baseline, only RLP exhibited a significant reduction (p<0.03). No changes were observed in the other variables studied.	Short duration, small sample, no analysis of the IM, no PG, and no dietary control.	The most promising result was a reduction in RLP-p, after 8 weeks, with therapeutic potential for treating cardiovascular diseases.
	Intervention period: 8 weeks.				
	IG: 5.6 x 10^10^ CFU/day of *Saccharomyces boulardii.*				
Tonucci et al. (2017)^25^/Clinical trial.	45 people aged 35 to 60 years with type 2 DM.	To investigate the effects of consumption of fermented goat’s milk containing *Lactobacillus acidophilus* La-5, *Bifidobacterium animalis lactis* BB-12, glycemic control, lipid profile, inflammation, oxidative stress, and short-chain fatty acids.	LDL and TG increased in the PG, which indicates a protective effect of the probiotics in the IG. There were significant difference between groups in mean change in TC (p=0.04) and LDL (p=0.03).	Short duration, small sample, and no group that did not consume fermented milk.	Consumption of *Lactobacillus acidophilus* La-5 and *Bifidobacterium animalis lactis* BB reduced LDL-c and TC.
	Intervention period: 6 weeks.				
	IG: 120 g/day of milk fermented with probiotics (*Lactobacillus acidophilus* La-5, *Bifidobacterium animalis lactis* BB-12; 10^9^ CFU of each).				
	PG: 120 g/day of conventional fermented milk with *Streptococcus thermophilus* TA-40.				

Apo A-V: apolipoprotein A-V; TC: total cholesterol; DM: diabetes mellitus; IG: intervention group; PG: placebo group; HDL-c: high density lipoprotein – cholesterol; BMI: body mass index; LDL-c: low density lipoprotein – cholesterol; IM: intestinal microbiota; OX-LDL: oxidative low density lipoprotein; RLP: remnant lipoproteins; TG: triglyceride; CFU: colony forming units.

The genera most often administered to groups treated with probiotics were *Lactobacillus*, in 11 studies, and *Bifidobacterium*, in six studies. Other genera used were *Saccharomyces*, *Streptococcus*, and *Enterococcus,* in one study each. The studies were designed to evaluate the effects of probiotics on the lipid profile and other variables. Some studies allocated subjects to groups treated with probiotics in isolation and in combination with other compounds, to enable observation of their effects with and without these additives.^14^^,^ 15 ^,^ 23 Kullisaar et al.^21^ did not form distinct groups to enable such comparisons. All of the clinical trials analyzed studied small samples, with a predominance of people with dyslipidemia, and were of short duration, varying from 15 days to 12 weeks.

## Discussion

Supplementation with probiotics significantly reduced total cholesterol (TC), LDL cholesterol (LDL-c),^13^^,^ 18 ^,^ 21 ^,^ 23 ^,^ 25 and triglycerides (TG)^12^^,^ 15 ^,^ 18 ^,^ 21 ^,^ 23 and increased HDL cholesterol (HDL-c).^15^^,^ 18 ^,^ 23 Other studies only observed significant effects when probiotics were combined with other treatments. Soy isoflavones and probiotics exhibited significant synergic effects that were not observed in groups given supplementation only.^14^ Additionally, physical exercise combined with administration of a fermented probiotics product stimulated an increase in HDL-c.^17^ Tonucci et al.^25^ found that only the control group exhibited increases in TC, LDL-c, and TG, suggesting that probiotics had a protective effect.

A meta-analysis of 11 articles detected reductions in TC and LDL in people with normal, borderline, and high cholesterol levels.^26^ Another meta-analysis found that probiotics had a positive effect on TC.^27^ Because the studies were highly heterogeneous, the authors also conducted an analysis of subsets and concluded that a group with TC higher than 200 mg/dL had the best response to treatment with probiotics.^27^ Subjects with hypertriglyceridemia also exhibited greater reductions in lipids.^12^ Ivey et al.^20^ explained that their results for lipid profile did not exhibit significant differences because the initial TC levels were lower than in other studies.

Other variables that are less frequently related to the lipid profile were also studied. Exposure of LDL-c to oxidative agents has been demonstrated in the atherosclerosis process, producing particles of oxidized LDL (oxLDL) and electronegative LDL.^28^ After consumption of a fermented soy product containing isoflavones, hypercholesterolemic individuals exhibited significant reductions in electronegative LDL.^14^ Findings reported by Fuentes et al.^18^ and Kullisaar et al.^21^ showed improvements in oxLDL. It has been observed that remnant lipoproteins (RLP) have a high atherogenic potential^29^ and it was suggested that *Saccharomyces boulardii* reduced the quantity of RLP.^24^

Several different factors affect the time needed to benefit the host’s lipid profile. At 15 days, significant improvement in the lipid profile were only observed when koumiss (a fermented product made with mare’s milk) was combined with physical exercise.^17^ The effectiveness of physical training for prevention and control of dyslipidemia has been well established.^30^ Childs et al.^15^ observed a significant increase in HDL-c after 15 days of a synbiotic, when compared with placebo. In a study by Kullisaar et al.,^21^ beneficial effects were observed after a 4-week intervention, but it cannot be confirmed whether *L. fermentum ME-3* is effective in isolation, because it was used with other compounds.

The estimated time needed to observe more definite results using probiotics in isolation appears to be 6 weeks.^23^^,^ 25 However, Fuentes et al.^18^ only observed significant differences between groups at 12 weeks and they were not observed at the halfway point of the study. Additionally, it was also observed that a 4-week interval without treatment, after 12 weeks of administration, reduced the beneficial effects on the lipid profile, although they were nevertheless still greater than in the placebo group.^18^ Therefore, continuous supplementation appears to be preferable, although if it is discontinued for up to 1 month, the desired effect may still be achieved.

Significant changes in anthropometric measurements were observed. Gomes et al.^19^ observed additional benefits from probiotics for waist circumference, conicity index, and waist-height ratio compared with a group that was only treated with a diet. A reduction in body mass index (BMI) was observed by Bernini et al.,^13^ in relation to baseline values.

Associations between obesity, metabolic disorders, and inflammation have been demonstrated in the literature.^31^ Improvement in inflammatory markers was demonstrated in some of the studies reviewed. Patients with type 2 Diabetes mellitus (DM2) who consumed fermented milk without probiotics exhibited reductions in interleukin-10 (IL-10) (p=0.001) and a tendency to reductions in adiponectin (p=0.07), both of which have anti-inflammatory functions. This reduction was not observed in a group that did consume probiotics, providing evidence that supplementation had a protective effect.^25^ Serum ultrasensitive C-reactive protein (PCR-us) and interleukin-6 (IL-6) levels were reduced in healthy volunteers (p<0.05).^21^ Over 45 days of treatment with probiotics, Bernini et al.^13^ observed significant reductions in tumor necrosis factor alpha (TNF-α) and IL-6. A study that employed a larger dose reported a significant reduction in PCR-us with probiotic use, whereas a group that consumed omega-3 did not exhibit the same impact.^23^ Healthy IM attenuates translocation of lipopolysaccharides (LPS), inducing lower numbers of inflammatory cytokines. However, the benefits observed were not associated with improvements in the inflammatory profile. The authors suggested that patients with higher levels of inflammation would exhibit the best results.^19^

A systematic review of 11 articles concluded that treatment with probiotics aids in treatment of DM2, improving many parameters related to glycemic control.^32^ In the present study, it was observed that Tonucci et al.^25^ reported a tendency for reductions in glycated hemoglobin (HbA1c) (p=0.06), compared with a control group, Kullisaar et al.^21^ observed significant improvement in HbA1c, and Rajkumar et al. found that fasting glycemia and insulin levels were reduced in groups given supplementation with probiotics, omega-3, and both probiotics and omega-3 (p<0.05).^23^

Additional effects, beyond the study objectives, were also observed. Oxidative mechanisms are involved in the processes leading to CVDs.^33^ Therefore, improvements in antioxidant defenses prevent damage to macromolecules and also endothelial injury. A significant increase in glutathione peroxidase (GPx) activity was observed in a group given probiotics, in comparison with a groups that only followed a diet. This effect was not observed in relation to superoxide dismutase (SOD).^19^

Individuals in a probable state of immunosenescence exhibited a positive effect on natural killer (NK) cell activity.^16^ It has also been suggested that probiotics and prebiotics have a modulating effect on Th1 and Th2 lymphocytes, helping those with low Th1 activation and reducing Th2 hyperactivation in atopic diseases.^15^

Studies reported dosages varying from 10^9^ to 112 × 10^9^ CFU/day. After 6 weeks, the largest dose demonstrated several beneficial effects and no adverse clinical effects were observed, suggesting that larger doses are safe.^23^

In order to benefit the host, probiotics must have the capacity to adhere to the intestinal mucosa, overcoming the barriers imposed by the gastrointestinal tract, primarily the gastric pH, bile salts, and pancreatic enzymes.^34^ In view of this, some studies tested for microorganisms in the intestine.^14^^,^ 15 ^,^ 23 ^,^ 25

Evidence of the effects of probiotics show that they are strain-dependent,^35^ indicating that generalization of the benefits of supplementation with probiotics should be treated with caution. As such, combining different strains appears to offer better results.^23^

The mechanisms possibly involved may act in a synergic manner to improve the lipid profile. Production of bile salt hydrolases by several genera causes deconjugation of the bile salts, with lower capacity for absorption and enterohepatic recirculation and increased excretion, in addition to increased demand for cholesterol to synthesize new molecules to replace losses. Deconjugation also alters the capacity do solubilize cholesterol, reducing absorption. Furthermore, cholesterol is incorporated into cell membranes during growth of the microorganisms. Another factor described in the literature is inhibition of hepatic synthesis of cholesterol and fatty acids through production of short-chain fatty acids.^11^^,^ 36

The overall panorama indicates that the capacity for reduction of serum lipids is limited compared to treatment with statins.^37^ However, probiotics offer a range of benefits, in addition to those already mentioned in this study, interacting both directly and indirectly, and possibly producing results of greater magnitude in relation to metabolic conditions and quality of life over the long term.^34^^,^ 35 ^,^ 36 The divergent results of different studies may be the result of the specificity and combination of the strains employed, the doses administered, the duration of the studies, and other extraneous variables.

## Conclusions

The scientific evidence indicates that there are relationships between IM and several health-related markers. Modulation of the IM with probiotics has yielded promising results. The majority of the clinical trials analyzed demonstrated that treatment with probiotics had a beneficial influence on the lipid profile. Additionally, improvements were also observed in inflammatory profile, glycemic control, body mass, and immunological markers, which are considered risk factors for development of CVDS.

This study indicates that supplementation with probiotics, as investigated in well-controlled studies, can be used as an adjuvant to traditional treatments for dyslipidemia. It is recommended that further studies be conducted, designed to identify the long-term effects and the influence of probiotics when used in combination with drug-based treatment.
