# Supplementary material for: Effects of probiotics on the lipid profile: systematic review
Source: J Vasc Bras. 2019 Aug 9;18:e20180124. doi: 10.1590/1677-5449.180124 (PMC6690648; doi:10.1590/1677-5449.180124)
Supplement: Translation

# Translation: Efeitos dos probióticos no perfil lipídico: revisão sistemática

**Authors:** Carlos Jorge Maciel Uchoa Gadelha, Alane Nogueira Bezerra

**Affiliations:** 1 Faculdade Metropolitana da Grande Fortaleza – FAMETRO, Fortaleza, CE, Brasil.; 2 Universidade Federal do Ceará – UF, Programa de Pós-graduação em Ciências Médicas, Fortaleza, CE, Brasil.

**Keywords:** dislipidemias, hipercolesterolemia, probióticos

## Abstract

As alterações na microbiota intestinal podem modular mecanismos envolvendo fatores de risco para doenças cardiovasculares, incluindo as dislipidemias. O objetivo foi revisar os efeitos da suplementação de probióticos na prevenção e no tratamento de alterações do perfil lipídico. As pesquisas foram feitas na base de dados PubMed, com os descritores “probiotics and lipid profile” e “probiotics and dyslipidemia”, em artigos publicados entre 2013 e 2018. A suplementação com probióticos reduziu significativamente o colesterol total, o colesterol LDL (lipoproteína de baixa densidade) e os triglicerídeos, assim como aumentou o colesterol HDL (lipoproteína de alta densidade). Alguns benefícios foram observados sobre variáveis antropométricas, de controle glicêmico, de estresse oxidativo, de inflamação e do sistema imune. O presente estudo sugere que a suplementação de probióticos seja indicada como tratamento coadjuvante às dislipidemias. Novos estudos devem ser desenvolvidos com a finalidade de esclarecer os efeitos de longo prazo, assim como a influência dos probióticos em associação com o tratamento medicamentoso.

## Introdução

O processo de urbanização e a consequente mudança no estilo de vida contribuíram para o aumento substancial de doenças crônicas, principalmente as doenças cardiovasculares (DCVs). Essas são a maior causa de mortes no mundo, gerando grandes prejuízos econômicos e no setor da saúde^1^. O acúmulo de gordura, principalmente visceral, através de complexas interações genéticas e ambientais está associado à inflamação sistêmica subclínica e aos diversos fatores de risco para o desenvolvimento de tais doenças^2^. Entre os fatores de risco, a hipercolesterolemia, a hipertrigliceridemia, o aumento da concentração de lipoproteína de baixa densidade (*low-density lipoprotein*, LDL) e a redução dos níveis de lipoproteína de alta densidade (*high-density lipoprotein*, HDL) constituem alvos importantes para a prevenção de DCV^3^^,^ 4.

Considerada importante reguladora do estado metabólico, a microbiota intestinal (MI) exerce diversas reações bioquímicas^5^. Segundo Huttenhower et al.^6^, a MI está ligada diretamente à manutenção da saúde, seja intestinal ou sistêmica. Sua instabilidade, chamada disbiose, pode alterar diretamente o desenvolvimento e as complicações de várias doenças, especialmente as doenças crônicas não transmissíveis. Na síndrome metabólica e nas DCVs, situações em que as dislipidemias podem estar presentes, existem mudanças na composição e na função da MI.

Indivíduos hipercolesterolêmicos, quando comparados aos indivíduos controles, possuem uma menor diversidade bacteriana da MI. Além disso, observa-se também uma diferença no perfil desses microrganismos, o que sugere um possível papel da MI no desenvolvimento da hipercolesterolemia^7^. A manipulação da MI com probióticos resulta em vários benefícios ao hospedeiro^8^^,^ 9. Eles já são utilizados na medicina humana, tanto na prevenção quanto no tratamento de doenças, através do controle da MI^10^.

O efeito hipocolesterolêmico dos probióticos tem sido investigado *in vitro* e *in vivo*, e controvérsias foram observadas^11^. Assim, sob a necessidade e a importância de novos métodos terapêuticos para controle e melhora do perfil lipídico a fim de auxiliar no tratamento de dislipidemias e de outras doenças crônicas não transmissíveis, o objetivo deste estudo foi revisar os efeitos da suplementação de probióticos na prevenção e no tratamento de alterações do perfil lipídico.

## Métodos

Trata-se de uma revisão sistemática cuja pesquisa foi realizada na base de dados PubMed utilizando a combinação dos descritores “probiotics and lipid profile” e “probiotics and dyslipidemia”, no período de publicação entre janeiro de 2013 e março de 2018. Foram incluídos estudos do tipo ensaio clínico publicados na língua inglesa, com indivíduos de faixa etária acima de 18 anos. Os critérios de exclusão utilizados foram para estudos com gestantes e lactantes.

## Resultados

Foram encontrados 28 ensaios clínicos publicados na íntegra. Após a utilização dos critérios de exclusão para estudos realizados com gestantes e lactantes, a remoção de estudos duplicados e a leitura de títulos, resumos e artigos na íntegra, foram incluídos 14 ensaios clínicos. O fluxograma do método PRISMA (*Preferred Reporting Items for Systematic Reviews and Meta-Analyses*) foi utilizado para detalhar o processo de seleção dos artigos, conforme a Figura 1.

**Figura 1:** Fluxograma da seleção de artigos para a revisão.

Os artigos incluídos analisaram a influência dos probióticos no perfil lipídico, conforme descrito no Tabela 1 12 ^-^ 25. Entretanto, nem todos os estudos avaliaram esses marcadores de maneira primária^15^^,^ 16 ^,^ 17 ^,^ 22.

**Tabela 1:** Descrição dos artigos.

**Autores/Tipo de estudo**	**Descrição da amostra**	**Objetivos**	**Resultados**	**Limitações**	**Conclusão**
Ahn et al. (2015)^12^/Ensaio clínico.	121 indivíduos com hipertrigliceridemia, não diabéticos.	Investigar o efeito da suplementação de duas cepas, *Lactobacillus curvatus HY7601* e *L. plantarum KY1052*, na capacidade de redução dos TG em indivíduos não diabéticos com valores limítrofes e moderados para hipertrigliceridemia.	Entre os grupos, houve redução de 18,3% de TG e de 15,6% no tamanho da partícula de LDL e aumento de apo A-V de 21,1% (p*<*0,05*).* Os valores de TG e apo A-V se correlacionaram inversamente.	Curta duração, amostra reduzida, ausência do controle da dieta e da caracterização da MI.	Os autores concluíram que a suplementação das cepas reduziu os níveis séricos do TG, sendo o maior efeito observado nos sujeitos com maiores níveis.
	Tempo de intervenção: 12 semanas.				
	GI: 2 gramas de pó contendo *Lactobacillus curvatus HY7601* e *L. plantarum KY1052*, 0,5 × 10^10^ UFC de cada.				
	GP: 2 gramas de um pó, sem microrganismos.				
Bernini et al. (2016)^13^/Ensaio clínico.	51 indivíduos entre 18 e 60 anos com síndrome metabólica.	Avaliar a influência de leite fermentado com *Bifidobacterium lactis HN019* no perfil lipídico, controle glicêmico e perfil inflamatório em pacientes com síndrome metabólica.	Foi observada uma diminuição significativa do CT (p=0,009) e LDL-c (p=0,008).	Curta duração, amostra reduzida, ausência da caracterização da MI.	Os dados mostraram potenciais efeitos do *Bifidobacterium lactis HN019* na redução de lipídios no sangue.
	Tempo de intervenção: 45 dias. GI: 26 indivíduos, consumindo leite fermentado com 2,72 × 10^10^ UFC de *Bifidobacterium lactis HN019*.				
	GP: sem intervenção.				
Cavallini et al. (2016)^14^/Ensaio clínico.	49 indivíduos do sexo masculino, de 45 a 48 anos, hipercolesterolêmicos.	Investigar a influência do produto de soja fermentado com *Enterococcus faecium CRL 183* e *Lactobacillus helveticus 416* mais isoflavonas.	Apenas os sujeitos do G1 tiveram redução significativa no CT. Quando comparado com o G2, resultou, no período final do estudo, na redução do LDL-c (p<0,05), no grupo de ambos os tratamentos, entre os dias 30 e 42, e a relação CT/HDL.	Curta duração, amostra reduzida, ausência da caracterização da MI.	Consumo regular dos probióticos *Enterococcus faecium CRL 183* e *Lactobacillus helveticus 416* contribuiu para a redução de marcadores de risco cardiovascular em homens hipercolesterolêmicos, através da melhora no perfil lipídico e diminuição da oxidação das partículas de LDL.
	Tempo de intervenção: 42 dias.				
	G1: 200 mL de produto fermentado de soja com probiótico *Enterococcus faecium CRL 183* e *Lactobacillus helveticus 416*, suplementado com isoflavona.				
	G2: 200 mL de produto de soja sem fermentação.				
	G3: 200 mL de produto fermentado de soja sem suplementação.				
Childs et al. (2014)^15^/Ensaio clínico.	43 sujeitos, idade entre 25 e 65 anos, saudáveis.	Verificar os efeitos do xylo-oligosaccharide e do *Bifidobacterium animalis BI–07* na MI e na função imune.	Foram verificados níveis de HDL maiores entre os indivíduos que receberam xylo-oligosaccharide, sem nenhuma diferença nos outros lipídios plasmáticos observados.	Curta duração, amostra reduzida.	Os dados indicam potenciais benefícios do xylo-oligosaccharide e do *Bifidobacterium animalis BI–07* na capacidade de aumentar o HDL.
	Submetidos a quatro tratamentos distintos (G1: maltodextrina; G2*: Bifidobacterium animalis BI–07*, 10^9^ UFC; G3: xylo-oligosaccharide, 8 g/d; G4: *Bifidobacterium animalis BI–07*, 10^9^ UFC + xylo-oligosaccharide, 8 g/d).				
	Tempo de intervenção: 21 dias, seguidos de 28 dias de *washout*.				
Dong et al. (2013)^16^/Ensaio clínico.	30 indivíduos saudáveis entre 55 e 74 anos.	Testar os probióticos no sistema imune em indivíduos com imunossenescência.	Os dados indicam que não teve diminuição significativa no CT e TG.	Curta duração, amostra reduzida, ausência de dados referentes ao perfil lipídico, ausência da caracterização da MI.	O consumo de probióticos *Lactobacillus casei Shirota* não apresentou efeito significativo no perfil lipídico.
	Tempo de intervenção: 4 semanas.				
	GI: *Lactobacillus casei shirota* com 1,3 x 10^10^ UFC/dia.				
	GP: leite desnatado sem o probiótico.				
Dönmez et al. (2014)^17^/Ensaio clínico.	18 indivíduos do sexo masculino, sedentários, com média de 33,66 anos de idade.	Investigar os efeitos do *koumiss* como um probiótico associado com exercício físico em variáveis hematológicas e bioquímicas em indivíduos sedentários.	Os níveis de CT e TG tenderam a diminuir em todos os grupos, mas apenas o G2 no dia 15. O HDL aumentou em todos os grupos, sendo o maior efeito observado no G2.	Curta duração, amostra reduzida, ausência da caracterização da MI.	Os achados sugerem que o *koumiss* com o exercício físico promove aumento do HDL.
	Tempo de intervenção: 15 dias.				
	G1: *koumiss*;				
	G2: *koumiss* mais exercício físico.				
	G3:exercício físico.				
	Principais probióticos para fermentação do *koumiss: L. delbrueckii* subsp. *Bulgaricus, L. salivarus, L. buchneri, L. plantarum, L. casei, L. helveticus, L. fermentum.*				
Fuentes et al. (2013)^18^/Ensaio clínico.	60 sujeitos hipercolesterolêmicos, de 18 a 65 anos.	Avaliar os efeitos da fórmula AB-LIFE® na concentração de lipídios e outros parâmetros relacionados ao risco cardiovascular em sujeitos hipercolesterolêmicos.	O GI diminuiu significativamente o CT e levou a uma tendência de redução de LDL-c e OX-LDL em comparação ao GP. O GI reduziu significativamente (p<0,05) CT, LDL-C, relação LDL-C: HDL-C e OX-LDL (13,6%, 14,7%, 19,7% e 13,6%, respectivamente) em relação à base. O aumento no HDL-C (p<0,05) foi observado apenas no GI.	Curta duração, amostra reduzida e ausência de caracterização da MI.	A suplementação de *Lactobacillus plantarum CECT* (*7527, 7528* e *7529*) contribuiu significativamente para a redução do colesterol sérico em pacientes hipercolesterolêmicos, apresentando melhor efeito naqueles com altos níveis de colesterol.
	IMC: 19-30 kg/m^2^.				
	Tempo de intervenção: 12 semanas.				
	GI: 1 cápsula de *Lactobacillus plantarum CECT (7527, 7528 e 7529*) com dose de 1,2 x 10^9^ UFC.				
	GP: 1 cápsula sem bactéria.				
Gomes et al. (2017)^19^/Ensaio clínico.	43 mulheres, entre 20 e 59 anos, com IMC entre 24-40 kg/m^2^.	Investigar se um mix de probióticos tem efeitos adicionais quando comparada a uma intervenção dietética isolada sobre a composição corporal, perfil lipídico, endotoxemia, inflamação e perfis antioxidante e anti-inflamatório.	Não houve diferença entre os grupos do LDL-c, apenas redução no resultado dos ácidos graxos poli-insaturados (GP= -5,65% *vs.* GI = - 18,63%, com p<0,04).	Curta duração, amostra reduzida e ausência de caracterização da MI.	A suplementação com o mix de probióticos teve efeito adicional quando comparada ao grupo com intervenção dietética apenas.
	Tempo de intervenção: 8 semanas.				
	GI: intervenção dietética mais mix de *Lactobacillus acidophilus*e, *L. casei*; *Lactococcus lactis*; *Bifidobacterium bifidum* e *lactis* com dose de 2 × 10^10^.				
	GP: Apenas intervenção dietética.				
Ivey et al. (2015)^20^/Ensaio clínico.	156 indivíduos com síndrome metabólica e idade média de 67 anos.	Determinar o efeito do *Lactobacillus acidophilus La5* e *Bifidobacterium animalis* subespécie *lactis Bb12* em forma de iogurte ou cápsula na pressão sanguínea e no perfil lipídico em homens e mulheres com síndrome metabólica.	Não houve alteração nos marcadores de perfil lipídico entre os grupos (p<0,05).	Curta duração, amostra reduzida, ausência da caracterização da MI.	As cepas de probióticos *Lactobacillus acidophilus La5* e *Bifidobacterium animalis* subespécie *lactis Bb12* não influenciaram as alterações de parâmetros do perfil lipídico, provavelmente devido aos valores de colesterol na base serem relativamente adequados.
	Tempo de intervenção: 6 semanas.				
	G1: iogurte mais cápsula placebo; G2: cápsula probiótica mais leite; G3 e G4: placebos.				
	Os indivíduos que consumiram probiótico ingeriram pelo menos 3 x 10^9^ UFC/dia.				
	Utilizaram o *Lactobacillus acidophilus La5* e *Bifidobacterium animalis* subespécie *lactis Bb12.*				
Kullisaar et al. (2016)^21^/Ensaio clínico.	45 indivíduos saudáveis entre 50 e 75 anos, com IMC entre 24-30 kg/m^2^, com fatores de risco limítrofe para doença cardiovascular.	Estabelecer se a formulação especial *Reg’active Cholesterol*® apresenta efeito positivo no sistema cardiovascular, perfis lipídico e inflamatório e hemoglobina glicada.	Houve redução significativa em todos os participantes de LDL-c, CT, TG e OX-LDL (p<0,05) e uma tendência de melhora no HDL.	Curta duração, ausência de grupo controle, amostra reduzida, ausência do controle dietético antes e após a intervenção, ausência da caracterização da MI, utilização de fórmula composta com vários nutrientes.	O consumo da fórmula em indivíduos com valores limítrofes para risco cardiovascular por um período de 4 semanas apresentou efeito positivo na redução de LDL-c, CT, TG e OX-LDL.
	Tempo de intervenção: 4 semanas.				
	GI: 2 cápsulas por dia contendo o probiótico *L. fermentum* ME-3 (6 × 10^9^ UFC/dia) junto a outros compostos.				
Ogawa et al. (2014)^22^/Ensaio clínico.	20 indivíduos com idade média de 51,1 anos e hipertrigliceridemia.	Examinar os efeitos do probiótico *Lactobacillus gasseri* SBT2055 (LG 2055) na resposta pós-prandial nos lipídios sanguíneos em japoneses com hipertrigliceridemia.	Com relação aos parâmetros de jejum, apenas os ácidos graxos não esterificados apresentaram diminuição significativa (p<0,01). Não houve diferença significativa no TG, CT, HDL e LDL.	Curta duração, amostra reduzida, ausência da caracterização da MI.	O consumo de probiótico reduziu os níveis de ácidos graxos não esterificados após sobrecarga oral de gordura e no período pós-ingestão de probiótico após 4 semanas do consumo do microrganismo.
	Tempo de intervenção: 4 semanas.				
	GI: 5 × 10^10^ UFC/dia de *Lactobacillus gasseri* SBT2055 (LG 2055), com frequência de 2 vezes ao dia.				
Rajkumar et al. (2014)^23^/Ensaio clínico.	60 indivíduos com sobrepeso, entre 40 e 60 anos.	Investigar se somente os probióticos ou em associação com o ômega 3 exercem alguma melhora no perfil lipídico, sensibilidade da insulina e resposta inflamatória em indivíduos com sobrepeso saudáveis.	G1: o HDL aumentou em 18,5% (p<0,01), e os valores de LDL (p<0,05), TG e VLDL (p<0,01) diminuíram em 7,04%, 5,8% e 12,98, respectivamente.	Curta duração, amostra reduzida, dificuldade de apontar efeito de alguma cepa específica.	Foram encontrados aumento do HDL e diminuição de TG, LDL e VLDL, sendo os melhores resultados encontrados no G3.
	Tempo de intervenção: 6 semanas.				
	G1: 112,5 x 10^9^ UFC com: *Bifidobacterium longum, Bifidobacterium infantis, Bifidobacterium breve, Lactobacillus acidophilus, Lactobacillus paracasei, Lactobacillus delbrueckii subespécie bulgaricus e Lactobacillus plantarum, e Streptococcus salivarius subespécie thermophilus.*		G2: aumentou o HDL em 23,2% e diminuiu o LDL em 10,7%, TG em 7,78% e VLDL em 7,78% (p<0,01).		
	G2: ômega 3:1, cápsula de 180 mg de EPA e 120 mg de DHA.				
	G3: probióticos e ômega 3.		G3: reduziu CT, TG e LDL, VLDL e aumentou o HDL em 6,7% (p<0,01).		
Ryan et al. (2015)^24^/Ensaio clínico.	11 homens hipercolesterolêmicos, de 21 a 69 anos.	Coletar evidências sobre o efeito do probiótico *Saccharomyces boulardii* no perfil lipídico e outros marcadores em adultos hipercolesterolêmicos.	Em comparação com a base, apenas a RLP apresentou redução significativa (p<0,03). Não foram observadas alterações nas demais variáveis estudadas.	Curta duração, amostra reduzida, ausência da caracterização da MI, ausência de GP e controle dietético.	O resultado mais promissor foi a diminuição da RLP-p, após 8 semanas, com potencial terapêutico para tratar doenças cardiovasculares.
	Tempo de intervenção: 8 semanas.				
	GI: 5,6 × 10^10^ UFC/dia de *Saccharomyces boulardii.*				
Tonucci et al. (2017)^25^/Ensaio clínico.	45 indivíduos entre 35 e 60 anos com DM tipo 2.	Investigar os efeitos da ingestão do leite de cabra fermentado contendo L*actobacillus acidophilus* La-5, *Bifidobacterium animalis lactis* BB-12, controle glicêmico, perfil lipídico, inflamação, estresse oxidativo e ácidos graxos de cadeia curta.	O GP aumentou o LDL e TG, o que caracteriza um efeito protetor dos probióticos no GI. Houve diferença significativa entre os grupos quanto às alterações médias de CT (p=0,04) e de LDL (p=0,03).	Curta duração, amostra reduzida, ausência do grupo que não consumiu leite fermentado.	O consumo de *Lactobacillus acidophilus* La-5 e de *Bifidobacterium animalis lactis* BB reduziu LDL-c e CT.
	Tempo de intervenção: 6 semanas.				
	GI: 120 g/dia de leite fermentado com probiótico (*Lactobacillus acidophilus* La-5, *Bifidobacterium animalis lactis* BB-12; 10^9^ UFC/de cada*)*.				
	GP: 120 g/dia de leite fermentado convencional com *Streptococcus thermophilus* TA-40.				

Apo A-V: apoliproteína A-V; CT: colesterol total; DM: diabetes melito; GI: grupo intervenção; GP: grupo placebo; HDL-c: lipoproteína de alta densidade – colesterol; IMC: índice de massa corporal; LDL-c: lipoproteína de baixa densidade – colesterol; MI: microbiota intestinal; OX-LDL: lipoproteína de baixa densidade oxidativa; RLP: lipoproteínas remanescentes; TG: triglicerídeo; UFC: unidade formadora de colônia.

Os gêneros mais utilizados nos grupos tratados com probióticos foram *Lactobacillus* com 11 estudos e *Bifidobacterium* com seis estudos. Utilizaram também os gêneros *Saccharomyces*, *Streptococcus* e *Enterococcus,* com apenas um estudo cada. Os estudos propuseram avaliar os efeitos dos probióticos sobre o perfil lipídico e outras variáveis. Alguns alocaram indivíduos em grupos de tratamento com os probióticos isolados e com adição de outros compostos, possibilitando a visualização dos efeitos com e sem sua adição^14^^,^ 15 ^,^ 23. Já Kullisaar et al.^21^ não apresentaram grupos distintos para essa comparação. Dos ensaios clínicos analisados, todos apresentaram amostra reduzida, com predominância de indivíduos dislipidêmicos, e curta duração, variando de 15 dias até 12 semanas.

## Discussão

A suplementação com probióticos reduziu significativamente o colesterol total (CT), o colesterol LDL (LDL-c)^13^^,^ 18 ^,^ 21 ^,^ 23 ^,^ 25 e os triglicerídeos (TG)^12^^,^ 15 ^,^ 18 ^,^ 21 ^,^ 23 e aumentou o colesterol HDL (HDL-c)^15^^,^ 18 ^,^ 23. Outros estudos observaram efeitos significativos apenas na associação dos probióticos com outro tratamento. As isoflavonas de soja e os probióticos apresentaram efeitos sinérgicos significativos, não observados nos grupos com a suplementação isolada^14^. Além disso, a prática de exercício físico associada ao uso de produto fermentado com probióticos promoveu o aumento do HDL-c^17^. Tonucci et al.^25^ verificaram que apenas o grupo controle aumentou CT, LDL-c e TG, sugerindo um efeito protetor dos probióticos.

Uma metanálise de 11 artigos verificou uma redução de CT e LDL em indivíduos com níveis normais, limítrofes e altos de colesterol^26^. Outra metanálise verificou efeito positivo dos probióticos no CT^27^. Devido à grande heterogeneidade, os autores conduziram uma análise de subgrupos e concluíram que o grupo com CT maior que 200 mg/dL teve melhor resposta ao tratamento com probióticos^27^. Indivíduos com hipertrigliceridemia também apresentaram maior redução dos lipídios^12^. Ivey et al.^20^ justificaram que seus resultados não mostraram diferenças significativas quanto ao perfil lipídico devido aos níveis iniciais de CT serem considerados menores do que em outros estudos.

Outras variáveis menos comumente relacionadas ao perfil lipídico também foram estudadas. A exposição de LDL-c a agentes oxidativos é verificada no processo de aterosclerose, produzindo partículas de LDL oxidada (oxLDL) e LDL eletronegativa^28^. Indivíduos hipercolesterolêmicos, após consumo de produto fermentado de soja com isoflavonas, reduziram de modo significativo a LDL eletronegativa^14^. Os achados de Fuentes et al.^18^ e Kullisaar et al.^21^ mostraram uma melhora na oxLDL. Observou-se que as lipoproteínas remanescentes (RLP) apresentaram alto potencial aterogênico^29^, sugerindo que o consumo de *Saccharomyces boulardii* reduziu o seu número^24^.

Diversos fatores influenciam o tempo necessário para beneficiar o hospedeiro em relação ao perfil lipídico. Com 15 dias, foi averiguada melhora significativa no perfil lipídico apenas com associação do *koumiss,* produto fermentado com leite de égua, à prática de exercício físico^17^. O treinamento físico já é bem reportado para prevenção e controle da dislipidemia^30^. Childs et al.^15^ visualizaram em 21 dias um aumento significativo no HDL-c com o simbiótico quando comparado ao placebo. No estudo de Kullisaar et al.^21^, foram observados efeitos benéficos com 4 semanas de intervenção, mas não é possível afirmar que o papel isolado do *L. fermentum ME-3* tenha efetividade devido à adição de outros compostos.

A estimativa de tempo necessário para visualização de resultados mais expressivos com o uso exclusivo de probióticos parece ser de 6 semanas^23^^,^ 25. Entretanto, Fuentes et al.^18^ visualizaram diferenças significativas entre os grupos apenas no período de 12 semanas, fato não observado na metade do estudo. Além disso, foi observado que um intervalo de 4 semanas de interrupção de tratamento, após 12 semanas de uso, diminuiu os efeitos benéficos no perfil lipídico, que ainda assim foram maiores que no grupo placebo^18^. Assim, a suplementação contínua parece favorável e, caso interrompida em até 1 mês, consegue surtir o efeito desejável.

Alterações significativas foram observadas em relação a medidas antropométricas. Gomes et al.^19^ encontraram benefício adicional dos probióticos sobre a circunferência da cintura, índice de conicidade e relação cintura-altura em comparação com o grupo que foi tratado apenas com a dieta. A redução do índice de massa corporal (IMC) foi observada por Bernini et al.^13^ em relação aos valores iniciais.

A associação entre obesidade, distúrbios metabólicos e inflamação é demonstrada na literatura^31^. A melhora de marcadores inflamatórios foi demonstrada em alguns estudos. Portadores de diabetes melito tipo 2 (DM2) que consumiram leite fermentado sem probióticos apresentaram redução de interleucina-10 (IL-10) (p=0,001) e tendência de redução de adiponectina (p=0,07), ambas com papel anti-inflamatório. Essa redução não foi observada no grupo que consumiu os probióticos, evidenciando efeito protetor com a suplementação^25^. Os níveis séricos de proteína C reativa ultrassensível (PCR-us) e interleucina-6 (IL-6) foram reduzidos em indivíduos saudáveis (p<0,05)^21^. Em 45 dias de tratamento com probióticos, Bernini et al.^13^ revelaram redução significativa no fator de necrose tumoral alfa (TNF-α) e IL-6. O estudo que utilizou maior dose reportou diminuição significativa na PCR-us com os probióticos, enquanto o grupo que consumiu ômega-3 não alcançou o mesmo impacto^23^. A MI saudável atenua a translocação de lipopolissacarídeos (LPS), induzindo menor número de citocinas inflamatórias. Entretanto, os benefícios encontrados não foram associados com a melhora do perfil inflamatório. Os autores sugerem que sujeitos expostos a maior grau de inflamação podem obter melhores resultados^19^.

Uma revisão sistemática com 11 artigos concluiu que o tratamento com probióticos auxilia no tratamento do DM2, melhorando diversos parâmetros relacionados ao controle glicêmico^32^. No presente estudo, observou-se que Tonucci et al.^25^ mostraram tendência de redução na hemoglobina glicada (HbA1c) (p=0,06) em comparação com o grupo controle. Kullisaar et al.^21^ observaram melhora significativa na HbA1c. A glicemia de jejum e os níveis de insulina foram reduzidos nos grupos com suplementação de probióticos, ômega-3 e ambos (p<0,05)^23^.

Efeitos adicionais, além dos objetivos do estudo, foram observados. Existem mecanismos oxidativos nos processos envolvidos na DCV^33^. Assim, a melhora nas defesas antioxidantes previne os danos gerados em macromoléculas, assim como a lesão endotelial. O incremento significativo da atividade da glutationa peroxidase (GPx) foi verificado no grupo que consumiu os probióticos em detrimento do que consumiu apenas a dieta. Isso não foi verificado com a superóxido dismutase (SOD)^19^.

Indivíduos em provável imunossenescência sofreram interferência positiva na atividade de células *natural killer* (NK)^16^. É sugerido um papel modulador do probiótico e do prebiótico estudados nos linfócitos Th1 e Th2, auxiliando aqueles com baixa ativação de Th1 e diminuindo a hiperativação de Th2 em doenças atópicas^15^.

Os estudos reportaram doses entre 10^9^ e 112 × 10^9^ UFC/dia. A maior dose demonstrou, em 6 semanas, vários efeitos benéficos sem a observância de alterações clínicas adversas, sugerindo o uso seguro para maiores doses^23^.

Para propiciar benefícios ao hospedeiro, os probióticos devem ter capacidade de aderir à mucosa intestinal, sobrepondo as barreiras impostas pelo trato gastrintestinal, principalmente o pH gástrico, os sais biliares e as enzimas pancreáticas^34^. Nessa perspectiva, alguns estudos avaliaram a presença dos microrganismos no intestino^14^^,^ 15 ^,^ 23 ^,^ 25.

Os efeitos evidenciados pelos probióticos são caracterizados como cepa-dependente^35^, indicando que a generalização dos benefícios dessa suplementação com probióticos deve ser feita com cautela. Assim, a combinação de diferentes cepas parece propiciar melhores resultados^23^.

Possíveis mecanismos podem atuar de forma sinérgica em favor da melhora do perfil lipídico. A produção de hidrolases de sais biliares por vários gêneros resulta em desconjugação dos sais biliares, em menor capacidade de absorção para recirculação entero-hepática e maior excreção, em requerimento aumentado do colesterol para síntese de novas moléculas a fim de repor a perda. A desconjugação também altera a capacidade de solubilização do colesterol, reduzindo a absorção. Além disso, ocorre incorporação do colesterol nas membranas celulares durante o crescimento dos microrganismos. A inibição da síntese hepática do colesterol e ácidos graxos através da produção de ácidos graxos de cadeia curta é outro fator descrito na literatura^11^^,^ 36.

O panorama indica capacidade limitada de redução dos lipídios séricos em comparação ao tratamento com estatinas^37^. Entretanto, os probióticos propiciam diversos benefícios, além dos supracitados neste estudo, interagindo de maneira direta e indireta, possivelmente promovendo resultados com maior magnitude em condições metabólicas e qualidade de vida em longo prazo^34^^,^ 35 ^,^ 36. A divergência entre os estudos pode ser oriunda da especificidade e combinação das cepas utilizadas, da dose administrada, da duração do estudo, além de outras variáveis intervenientes.

## Conclusão

As evidências científicas indicam relação da MI com vários marcadores relacionados à saúde. A modulação da MI com probióticos tem apresentado resultados promissores. A maioria dos ensaios clínicos analisados demonstrou influência benéfica no perfil lipídico através do tratamento com probióticos. Além disso, também foram observadas melhoras no perfil inflamatório, controle glicêmico, massa corporal e marcadores imunológicos, considerados fatores de risco para o desenvolvimento de DCV.

O presente estudo indica a suplementação de probióticos, previamente investigados em estudos bem controlados, como coadjuvante no tratamento tradicional das dislipidemias. Sugere-se a realização de estudos futuros com a finalidade de esclarecer os efeitos de longo prazo, assim como a influência dos probióticos em associação com o tratamento medicamentoso.
